# Dynamics of High-Risk Nonvaccine Human Papillomavirus Types after Actual Vaccination Scheme

**DOI:** 10.1155/2014/542923

**Published:** 2014-04-03

**Authors:** Raúl Peralta, Cruz Vargas-De-León, Augusto Cabrera, Pedro Miramontes

**Affiliations:** ^1^Departamento de Matemáticas, Facultad de Ciencias, UNAM. Avenida Universidad 3000, Circuito Exterior S/N Delegación Coyoacán, C.P. 04510 Ciudad Universitaria, D.F., Mexico; ^2^Centro de Ciencias de la Complejidad (C3), Torre de Ingeniería, UNAM. Avenida Universidad 3000, Circuito Exterior S/N Delegación Coyoacán, C.P. 04510 Ciudad Universitaria, D.F., Mexico; ^3^Hospital General de México, Dr Balmis No. 148. Col. Doctores C.P. 06726 México, D.F., Mexico; ^4^Unidad Académica de Matemáticas, Universidad Autónoma de Guerrero, Avenida Lázaro Cárdenas S/N, Ciudad Universitaria, C.P. 39090 Chilpancingo, GRO, Mexico

## Abstract

Human papillomavirus (HPV) has been identified as the main etiological factor in the developing of cervical cancer (CC). This finding has propitiated the development of vaccines that help to prevent the HPVs 16 and 18 infection. Both genotypes are associated with 70% of CC worldwide. In the present study, we aimed to determine the emergence of high-risk nonvaccine HPV after actual vaccination scheme to estimate the impact of the current HPV vaccines. A SIR-type model was used to study the HPV dynamics after vaccination. According to the results, our model indicates that the application of the vaccine reduces infection by target or vaccine genotypes as expected. However, numerical simulations of the model suggest the presence of the phenomenon called vaccine—induced pathogen strain replacement. Here, we report the following replacement mechanism: if the effectiveness of cross-protective immunity is not larger than the effectiveness of the vaccine, then the high-risk nonvaccine genotypes emerge. In this scenario, further studies of infection dispersion by HPV are necessary to ascertain the real impact of the current vaccines, primarily because of the different high-risk HPV types that are found in CC.

## 1. Introduction

SIR (susceptible, infected, and recovered) mathematical models are widely used to improve the understanding of disease dynamics [1]. These models usually include some epidemiological factors involved in the development of a disease, such as transmission rate and recovery, and in the case of infectious diseases, they incorporate the control schemes such as the application of prophylactic vaccines [2]. These models divide the population into susceptible, infected, and recovered classes and, in the case of sexually transmitted diseases, also consider the exposed class, which are those individuals who have had contact with the infectious agent but have not yet acquired the infection. With these factors or parameters, it is possible to build a mathematical SIR type model that allows us to predict the disease dynamics.

The use of SIR models in the study of the dynamics of infection by human papillomavirus (HPV) has helped to the understanding of the impact of vaccination schemes against oncogenic HPVs 16 and 18 infection and warts causing genital HPVs 6 and 11 types [3, 4]. A previous model [3, 4] studied HPV infection, disease progression, cervical cancer (CC) screening, treatment, vaccine characteristics, vaccination strategies, and the impact of HPV quadrivalent vaccination on epidemiological and economic outcomes within a US population. The main benefit is the reduction of HPVs 16, 18, 6, and 11 infection as expected. Consistent with these results reported for the US population, data from an analysis suggest that a quadrivalent HPV vaccine can yield substantial benefits in reducing the incidence of cervical cancer, cervical intraepithelial neoplasia (CIN), and genital warts in Mexico [4].

HPV is the main etiological factor for the development of cervical lesions and CC. The epidemiological evidences concluded that nearly 70% of CC could be attributed to HPV 16 (nearly 60%) and HPV 18 (nearly 10%). The associations with these HPV types are consistent in all studies and countries for CC. However, HPV genotypes are highly diverse; so far, nearly 150 HPVs have been described and more than 40 genotypes infect the anogenital tract. For twelve, there is sufficient evidence of their carcinogenicity; that is, they have the capacity to lead to cell transformation (HPVs 16, 18, 31, 33, 35, 39, 45, 51, 52, 56, 58, and 59) and have been classified as high-risk (hr) or carcinogenic to humans, while HPV types 6 and 11 are classified as low-risk (lr) or not classifiable as to their carcinogenicity to humans, causing benign epithelial proliferations [5]. Collectively, hr nontarget or nonvaccine HPVs contribute with nearly 30% of CC worldwide [6]. In this context, with the inclusion of a second generation of vaccines that protect against hr nonvaccine HPVs, it could protect almost entirely from the CC [5].

CC is the second most common cancer in women worldwide and the HPV infection is the major etiological factor for the development of this tumor. This infection is the most common sexually transmitted disease among sexually active men and women worldwide, and it has been estimated that at least 50% of sexually active individuals have had a genital HPV infection [7]. The prevalence of HPV infection varies worldwide, being higher in developing countries [6]. In clinically healthy Mexican women, the prevalence of HPV infection is 15% [8], a percentage similar to that found in other developing countries [6, 9, 10] and the prevalence of HPVs 16 and 18 infection in CC is 71% (63% for HPV 16 and 8% for HPV 18), while the prevalence of HPV infection with other genotypes is 25%, highlighting the hr HPVs 31 and 58 as the most frequent (5% for each type) [8]. In 2008, an estimated number of 5,061 Mexican women died as result of CC, making it one of the major causes of death in Mexican women [11]. It is expected that current vaccines directed against HPVs 16 and 18 will reduce the overall incidence of CC.

However, these vaccines could modulate positively the prevalence of infections with different genotypes through a replacement of viral genotype, which could lead to an increase in the prevalence of hr nonvaccine HPVs after actual vaccination scheme [12, 13]. In some studies, it has been reported that the vaccine could confer cross-protective immunity against related genotypes which would lead to a decrease in the transmission of these hr nonvaccine HPVs [13], but, on the other hand, the removal of some HPVs (HPVs 16 and 18 or vaccine HPVs) as the result of vaccination may result in a positive selection pressure with other hr HPVs or hr nonvaccine HPV types, leading to an increase in transmission of the latter. This phenomenon, known as vaccine-induced pathogenic strain replacement, was previously described in a vaccination program against other pathogens [14]. Such was the case of* Streptococcus pneumoniæ* in which the vaccine, directed against seven serotypes, resulted in an increase of other serotypes [15]. HPVs are highly stable DNA viruses; thus selective pressures from vaccination may vacate existing ecological niches currently taken by HPVs 16 and 18 [16].

In this study a mathematical SIR type model to predict the dynamic of hr nonvaccine HPVs infection after actual vaccination scheme is proposed. Thus, the identification of emerging high risk genotypes of nonvaccine HPVs must be incorporated into a second generation vaccine to cover a broader spectrum of HPV infection associated with CC.

## 2. Model Formulation

Our model considers two genotypes, one that includes high-risk 16 and 18 genotypes or hr-vaccine-type HPV and one which includes high-risk 31, 33, 35, 39, 45, 51, 52, 56, 58, and 59 genotypes or hr-nonvaccine-type HPV.

The total population at time *t*, denoted by *N*(*t*), is subdivided into mutually exclusive compartments for individuals that are unvaccinated susceptible (*S*(*t*)), vaccinated susceptible (*V*(*t*)), infected with vaccine-type HPV (*I*_1_(*t*)) and infected with nonvaccine-type HPV (*I*_2_(*t*)) so that

(1) $$\begin{array}{c} S\left(t\right)+V\left(t\right)+I_{1}\left(t\right)+I_{2}\left(t\right)=N\left(t\right). \end{array}$$

The unvaccinated susceptible population increases by the recruitment of new sexually active individuals (assumed susceptible) into the population (at a rate Λ) and the loss of protection of the vaccine (at a rate *γ*). This population diminishes by “sexual death” (at a rate *μ*), vaccination of new sexually active individuals (at a rate *ϕ*Λ), and the acquisition of HPV infections with nonvaccine genotypes and nonvaccine genotypes, following effective contact with infectious individuals (in the *I*_1_(*t*) and *I*_2_(*t*) classes), at rates *λ*_*s*1_ and *λ*_*s*2_, respectively, where

(2) $$\begin{array}{c} \lambda_{s1}=\frac{\beta_{1}I_{1}}{N},\;\;\lambda_{s2}=\frac{\beta_{2}I_{2}}{N} \end{array}$$

are the* force of infection* and *β*_1_ and *β*_2_ are the effective contact rates. Putting the above assumptions and definitions together gives the following equation for the rate of change of the unvaccinated susceptible population:

(3) $$\begin{array}{c} \frac{\text{d}S}{\text{d}t}=\left(1-\phi \right)\Lambda -\left(\lambda_{s1}+\lambda_{s2}\right)S-\mu S+\gamma V. \end{array}$$

The population of vaccinated susceptible individuals (*V*) increases due to the vaccination of new sexually active individuals (at the rate *ϕ*Λ). Further, this population diminishes by the “sexual death” (at the rate *μ*), rate at which the vaccine wanes *γ*, and the acquisition of HPV infections with vaccine genotypes and nonvaccine genotypes at rates *λ*_*v*1_ and *λ*_*v*2_, respectively, where

(4) $$\begin{array}{c} \lambda_{v1}=\frac{\epsilon \beta_{3}I_{1}}{N},\;\;\lambda_{v2}=\frac{\sigma \beta_{4}I_{2}}{N} \end{array}$$

are the* force of infection* and *β*_3_ and *β*_4_ are the effective contact rates. The vaccine has the effect of reducing the force of infection by a factor of 0 ≤ *ϵ* < 1, so that *ϵ* = 0 means the vaccine is completely effective in preventing HPV infection with vaccine genotypes. Thus 1 − *ϵ* is the vaccine efficacy. Some reports indicate that the vaccination confers partial cross-protective immunity to nonvaccine genotypes [13]. Partial cross-protective immunity is explored in terms of relative susceptibility to infection with nonvaccine genotypes, where *σ* can be any value between zero and one (0 < *σ* < 1). For example, a value of *σ* = 0.8 can be interpreted as 20% protection against infection with nonvaccine genotypes for vaccinated sexually active individuals. Thus,

(5) $$\begin{array}{c} \frac{\text{d}V}{\text{d}t}=\phi \Lambda -\lambda_{v1}V-\lambda_{v2}V-\left(\mu +\gamma \right)V. \end{array}$$

The population of individuals infected with vaccine-type HPV is generated by the infection of unvaccinated susceptible individuals (at the rate *λ*_*s*1_) and vaccinated susceptible individuals (at the rate *λ*_*v*1_). It is reduced by the development of cervical cancer associated with vaccine-type HPV infection (at a rate *η*_1_) and by natural recovery rate (at a rate *γ*_1_) and “sexual death” (at a rate *μ*). By letting *α*_1_ = *η*_1_ + *γ*_1_, equation can be represented as

(6) $$\begin{array}{c} \frac{\text{d}I_{1}}{\text{d}t}=\lambda_{s1}S+\lambda_{v1}V-\left(\mu +\alpha_{1}\right)I_{1}. \end{array}$$

The population of individuals infected with nonvaccine-type HPV is generated by the infection of unvaccinated susceptible individuals (at the rate *λ*_*s*2_) and the infection of vaccinated susceptible individuals with nontarget genotype is produced at a rate *λ*_*v*2_. It is reduced by the development of cervical cancer associated with nonvaccine-type HPV infection (at a rate *η*_2_) and by natural recovery rate (at a rate *γ*_2_) and “sexual death” (at a rate *μ*). By letting *α*_2_ = *η*_2_ + *γ*_2_, equation can be represented as

(7) $$\begin{array}{c} \frac{\text{d}I_{2}}{\text{d}t}=\lambda_{s2}S+\lambda_{v2}V-\left(\mu +\alpha_{2}\right)I_{2}. \end{array}$$

Thus, the basic model for the transmission dynamics of HPV in a population is given by the following system of nonlinear differential equations:

(8) dSdt=(1−ϕ)Λ−β1NSI1−β2NSI2−μS+γVdVdt=ϕΛ−ϵβ3NVI1−σβ4NVI2−(μ+γ)VdI1dt=β1NSI1+ϵβ3NVI1−(μ+α1)I1dI2dt=β2NSI2+σβ4NVI2−(μ+α2)I2.

We assume that all parameters are positive except *ϵ* which is nonnegative. Set the initial condition of system (8) as follows: *S*(0) = *S*_0_ > 0, *V*(0) = *V*_0_ > 0, *I*_1_(0) = *I*_01_ > 0, and *I*_2_(0) = *I*_02_ > 0.

## 3. Local Stability of HPV-Free Equilibrium and Numerical Simulations

The system (8) always has the HPV-free equilibrium *E* ^0^ = (*S* ^0^, *V* ^0^, 0,0), where

(9) $$\begin{array}{c} S^{0}=\frac{\left(1-\phi \right)\Lambda }{\mu }+\frac{\gamma }{\mu }V^{0}=\frac{\Lambda }{\mu \left(\mu +\gamma \right)}\left(\left(1-\phi \right)\left(\mu +\gamma \right)+\phi \gamma \right),\\ V^{0}=\frac{\phi \Lambda }{\mu +\gamma }. \end{array}$$

The vaccine reproduction number for the hr-vaccine-type HPV is

(10) Rv1=β1S0(μ+α1)(S0+V0)+ϵβ3V0(μ+α1)(S0+V0)=((1−ϕ)(μ+γ)+ϕγ)β1(μ+γ)(μ+α1)+ϕμϵβ3(μ+γ)(μ+α1).

This reproductive number is the average number of secondary HPV infected individuals produced by each infected individual with hr-vaccine-type HPV.

Similarly, we now define the vaccine reproduction number for the hr-nonvaccine-type HPV:

(11) Rv2=β2S0(μ+α2)(S0+V0)+σβ4V0(μ+α2)(S0+V0)=((1−ϕ)(μ+γ)+ϕγ)β2(μ+γ)(μ+α2)+ϕμσβ4(μ+γ)(μ+α2).

**Remark 1**

Under the following restrictions on the parameters *β* = *β*_1_ = *β*_2_ = *β*_3_ = *β*_4_ and *α* = *α*_1_ = *α*_2_, we note that if the vaccine efficacy 1 − *ϵ* is greater than the efficacy of partial cross-protective immunity 1 − *σ* then *R*_*v*_^2^ > *R*_*v*_^1^.

(12) $$\begin{array}{c} R_{v}^{2}-R_{v}^{1}=\frac{\phi \mu \beta \left(\sigma -\epsilon \right)}{\left(\mu +\gamma \right)\left(\mu +\alpha \right)}>0, \end{array}$$

it is epidemiologically feasible that *σ* > *ϵ*.

We now study the local stability behavior of the HPV-free equilibrium *E* ^0^ = (*S* ^0^, *V* ^0^, 0,0) for system (8). The local stability follows by analyzing the Jacobian matrix

(13) $$\begin{array}{c} J\left(E^{0}\right)=\left(\begin{array}{cccc} -\mu & \gamma & -\frac{\beta_{1}S^{0}}{S^{0}+V^{0}} & -\frac{\beta_{2}S^{0}}{S^{0}+V^{0}}\\ 0 & -\left(\mu +\gamma \right) & -\frac{\epsilon \beta_{3}V^{0}}{S^{0}+V^{0}} & -\frac{\sigma \beta_{4}V^{0}}{S^{0}+V^{0}}\\ 0 & 0 & A_{33}^{0} & 0\\ 0 & 0 & 0 & A_{44}^{0} \end{array}\right), \end{array}$$

where

(14) $$\begin{array}{c} A_{33}^{0}=\frac{\beta_{1}S^{0}}{S^{0}+V^{0}}+\frac{\epsilon \beta_{3}V^{0}}{S^{0}+V^{0}}-\left(\mu +\alpha_{1}\right),\\ A_{44}^{0}=\frac{\beta_{2}S^{0}}{S^{0}+V^{0}}+\frac{\sigma \beta_{4}V^{0}}{S^{0}+V^{0}}-\left(\mu +\alpha_{2}\right). \end{array}$$

The eigenvalues of *J*(*E* ^0^) are *τ*_1_^0^ = −*μ* < 0 and *τ*_2_^0^ = −(*μ* + *γ*) < 0, and

(15) $$\begin{array}{c} \tau_{3}^{0}=\frac{\beta_{1}S^{0}}{S^{0}+V^{0}}+\frac{\epsilon \beta_{3}V^{0}}{S^{0}+V^{0}}-\left(\mu +\alpha_{1}\right)=-\left(\mu +\alpha_{1}\right)\left(1-R_{v}^{1}\right),\\ \tau_{4}^{0}=\frac{\beta_{2}S^{0}}{S^{0}+V^{0}}+\frac{\sigma \beta_{4}V^{0}}{S^{0}+V^{0}}-\left(\mu +\alpha_{2}\right)=-\left(\mu +\alpha_{2}\right)\left(1-R_{v}^{2}\right). \end{array}$$

The eigenvalues *τ*_3_^0^ and *τ*_4_^0^ are negative if and only if *R*_*v*_^1^ < 1 and *R*_*v*_^2^ < 1. Therefore *E* ^0^ is locally asymptotically stable for *R*_*v*_^1^ < 1 and *R*_*v*_^2^ < 1 and unstable otherwise. Then we have the following theorem.

**Theorem 2**

If *R*_*v*_^1^ < 1 and *R*_*v*_^2^ < 1, *E* ^0^ = (*S* ^0^, *V* ^0^, 0,0) is locally asymptotically stable and unstable otherwise.

Based on the literature and our estimations, we consider a set of parameter values that may represent a plausible scenario, although they are not necessarily realistic as a whole. We assume that the time unit is* year* and estimate the parameters as follows.

- *Recruitment rate, *Λ. As reported in [17, 18] the recruitment rate of new sexually active individuals is Λ = 10000 year^−1^.
- *Sexual activity period, *1/*μ*. If we assume that the sexually active period of healthy individuals lies between 15 and 30 years [19], so that we take *μ* = 1/30  *years* = 0.0333 year^−1^.
- *Vaccination rate, ϕ*. The proportion of vaccinated new sexually active individuals (cohort vaccination) is *ϕ* = 0.7 year^−1^ [18, 20].
- *Loss rate of vaccine efficacy, γ*. The duration of vaccine protection is unknown. However recent studies show that the vaccine induces a strong immune response, with high and sustained levels of IgG and neutralizing antibodies against HPV-16/18 up to 7.3 years [21]. Studies expect protection to continue for many more years, so that we take *γ* = 1/10  *years* = 0.10 year^−1^.
- *Contact rate, β*_*i*_  (*i* = 1,2). This parameter is given by *β*_*i*_ = *bp*, where *b* is the average number of sexual contacts per person and *p* is the probability of successful HPV infection. As reported in [17, 18], the average number of sexual contacts per person is two, 2 *contact*/*year*, and *p* = 0.8/*contact*. Hence we take *β*_*i*_ = 1.6 year^−1^. We will assume that contact rates are equal.
- *Vaccine efficacy, *1 − *ϵ*. The efficacy of the vaccine may range from 90% to 100% [3, 22], so that we take 1 − *ϵ* = 0.9. Hence the value of *ϵ* is 0.1.
- *Partial cross-protective immunity, σ*. We choose, *σ* = 0.8, that is to say, 20% protection against infection with nonvaccine genotypes for vaccinated sexually active individuals.

Furthermore, we will assume that *α*_1_ and *α*_2_ are equal. We take *α*_1_ = *α*_2_ = 0.02916 year^−1^ (which means that 16 years are estimated as the average residence time 1/(*μ* + *α*_*i*_) in the infected class.)

Choosing these values, the reproductive numbers are as follows: *R*_*v*_^1^ = 21.568 < *R*_*v*_^2^ = 24.704. Three equilibrium points were calculated for the epidemic model (8). One equilibrium occurred when *S* ^0^ = 247500, *V* ^0^ = 52500, and *I*_1_^0^ = *I*_2_^0^ = 0, which we call HPV-free equilibrium *E* ^0^. The other equilibrium points are the hr-vaccine-type HPV equilibrium

(16) $$\begin{array}{c} E^{1}=\left(4168,26387,143703,0\right) \end{array}$$

and the hr-nonvaccine-type HPV equilibrium

(17) $$\begin{array}{c} E^{2}=\left(2253,5165,0,156043\right). \end{array}$$

**Figure 1:**
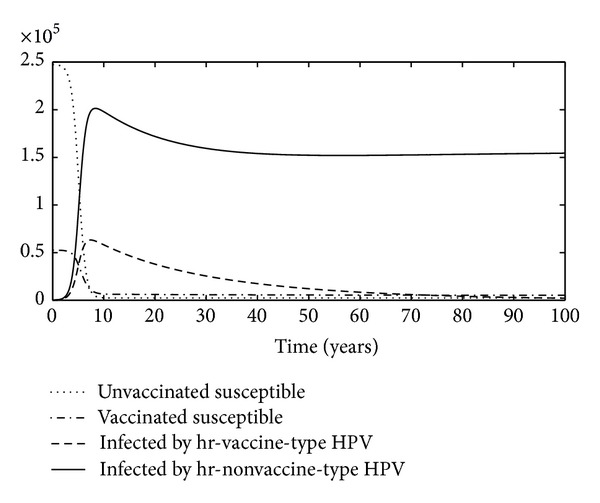
The graphs show unvaccinated susceptible, vaccinated susceptible, infected with vaccine-type HPV, and infected with nonvaccine-type HPV versus years.

The HPV-free equilibrium *E* ^0^ and the hr-vaccine-type HPV equilibrium *E* ^1^ are unstable, and the hr-nonvaccine-type HPV equilibrium *E* ^2^ is locally stable, as determined by the Jacobian matrix of the system (8) at an equilibrium (see Appendix). In Figure 1 the time evolution of the populations is plotted. It is shown that the equilibrium *E* ^2^ is asymptotically stable.

## 4. Discussion and Conclusions

It is already known that persistent hr HPV infection is widely associated with the development of CC [5]. This finding has propitiated the development of vaccines that help to prevent this infection. Particularly, epidemiological studies show that hr HPVs 16 and 18 infections are associated with 70% of CC worldwide. In contrast, the hr HPVs (31, 33, 35, 39, 45, 51, 52, 56, 58, and 59) infections are associated with nearly 30% of CC. These findings suggest a molecular mechanism involved in the cellular transformation mediated by hr HPV infection, on one hand, a group of highly oncogenic hr HPV and, on the other, a group of fewer oncogenic hr HPV. This observation supports previous strategies to prevent this infection, mediated by application of prophylactic vaccines.

SIR are currently being used to determine the reduction of this infection by the effect of the vaccine [22]. Such models explain the reduction of infection with hr HPVs 16 and 18 included as targets of the vaccine [4, 22, 23]. Hence the universal application of this vaccine has the potential to reduce the incidence of CC associated with this genotypes infection [23] and the decrease in HPVs 16 and 18 is also promising in terms of its potential impact on rates of anal, vulvae, and head and neck cancers because some of them are caused by one of these types [24]. Our model considers two genotypes: one that includes the hr HPVs 16 and 18 or vaccine HPV and other including hr HPVs 31, 33, 35, 39, 45, 51, 52, 56, 58, and 59 or nonvaccine HPV. The numerical simulations of our model indicate that the application of the vaccine declines infection by target genotypes as expected. However, our results on dynamics of nonvaccine HPV types infection show the possible presence of a biological mechanism: vaccine-induced pathogenic strain replacement, for example, an increase in the prevalence of HPV genotypes not targeted by the vaccines due to an ecological niche created by a reduction in the prevalence of HPV genotypes targeted by the vaccines. According to the ecological notion, a population will expand its niche once another population is removed from a shared environment [25]. In this context, Murall, McCann, and Bauch developed an intrahost mathematical model that represents HPV coinfections in epithelial tissues and the immune response to infection. This study reported that a type replacement remains viable if nonvaccine targeted types that are not cross-reactive with the vaccine could spread to more patches (or tissues) and can increase their viral load in vaccinated host [25]. This observation is documented for various pathogens [13, 15], such as the immunization against* Hæmophilus influenzæ* serotype *b* in the United States that resulted in the increase of serotype *f* in the new cases of influenza [13]. This phenomenon is also present in other pathogens of bacterial origin [15]; for example, a significant increase in the prevalence of nonvaccine serotype occurred after introduction of a heptavalent conjugate pneumococcal vaccine and a* Bordetella pertussis* vaccine [26]. In this context, our mathematical model correctly predicts the emergence of viral genotypes nontarget of the vaccine similar to the aforementioned phenomenon.

In order to define a possible mechanism of vaccine-induced pathogenic strain replacement, we attempted to more comprehensively examine hr nonvaccine HPV dynamic using the SIR epidemiological model. Interestingly, the dynamic of vaccine HPV infection showed a decrease as expected; in contrast, the dynamic of hr nonvaccine HPV infection showed an increase after actual vaccination scheme. In this regard, some studies have described that the vaccine could reduce nonvaccine HPV infection by a mechanism of cross-immunity; this is due to the high similarity of sequences in the L1 region in hr HPV [13]. Based on these sequences, the hr HPVs are divided into the group A7 (which includes the 18, 39, 45, 51, and 68 genotypes) and A9 (which includes the 16, 31, 33, 35, 52, and 58 genotypes) [27]. These HPV groups contain almost all hr HPVs causing CC, hence to prevent these infections would have the effect of reducing the CC [27]. On the other hand, HPV vaccination is expected to provide herd protection, that is, to provide indirect protection to those who have not been vaccinated, due to a reduced prevalence of HPV in communities [28]. Based on the present study, we can suggest that only the reduction of the HPVs 16 and 18 infection is possible for the effectiveness of the vaccine (90–100%) [22]; however, there is not a reduction of hr nonvaccine HPVs.

In the context of HPV infection the following statements should be adopted: (1) HPVs are highly stable DNA viruses; thus, selective pressure from vaccination may vacate existing ecological niches currently taken by HPVs 16 and 18 [16]. (2) The high prevalence of HPV infection and frequency on concurrent infections with more than one type provides an opportunity for HPV type interactions [29]; vaccination cannot remove this infection but prevents potential future infection with vaccine-HPVs [16]. (3) The HPV vaccination is likely to be beneficial to sexually active adult women as they are at risk of acquiring new HPV infection [30]. (4) According to basic ecological principles, if competition exists between two or more different HPV types for niche occupation during natural infection, elimination of one type might lead to an increase in other types [31]. Epidemiologic approaches for evaluation of HPV type competition has been revised [31].

**Figure 2:**
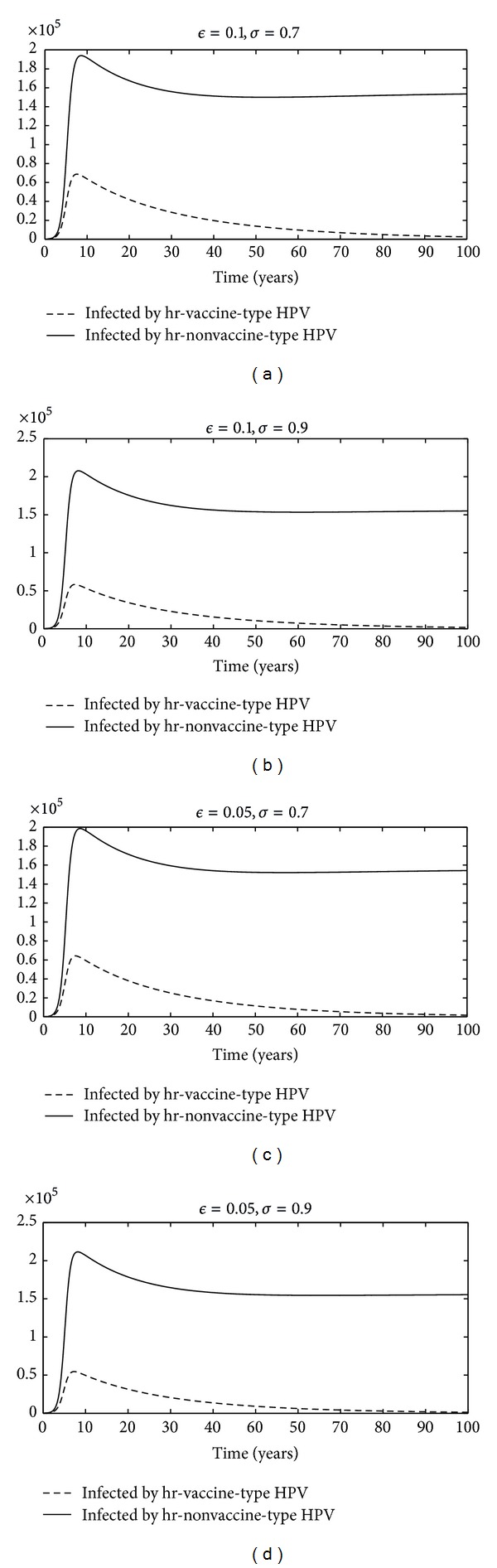
Time profile of infected individuals of system (8) corresponding to different pairs of values of *ϵ* and *σ*. These simulations suggest that if the effectiveness of cross-protective immunity is not larger than the effectiveness of the vaccine, then the high-risk nonvaccine-type HPV emerge.

**Figure 3:**
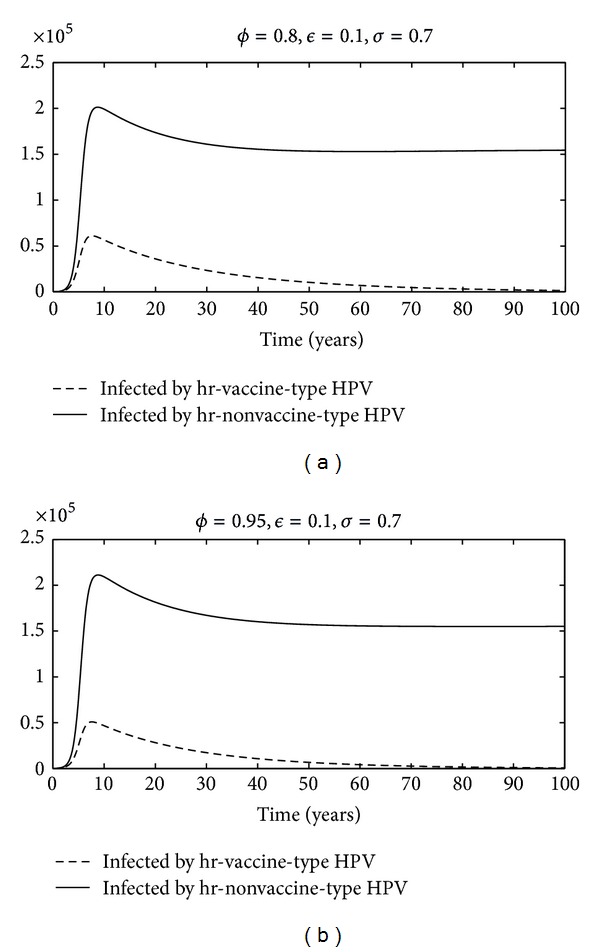
Time profile of infected individuals of system (8) corresponding to two parameter values of *ϕ*.

Here, we report an increased incidence of hr nonvaccine HPV after vaccination campaigns; the vaccine-induced pathogenic strain replacement occurs if the effectiveness of cross-protective immunity is not larger than the effectiveness of the vaccine (see Figures 2 and 3). In this scenario, further studies of spread infection by HPV are necessary to ascertain the real impact of the current vaccines, primarily because the different high risk HPV types are found in CC. According to the present analysis, a potential new-generation vaccine directed against groups A7 and A9 papillomavirus would protect more widely the population. This would provide optimal prevention of CC.

## Appendix

We explore the local stability of nonnegative equilibria of model (8) with the parameter values given in Section 3.

The HPV-free equilibrium *E* ^0^ = (247500,52500,0, 0) always exists. The eigenvalues for the HPV-free equilibrium described by Jacobian matrix *J*(*E* ^0^) were

(A.1) τ10=−0.03333333333<0,τ20=−0.1333333333<0,τ30=1.2855>0,τ40=1.4815>0.

The presence of two positive eigenvalues then the HPV-free equilibrium *E* ^0^ = (247500,52500,0, 0) is unstable.

Now we examine the local stability of hr-vaccine-type HPV equilibrium *E* ^1^ = (4168,26387,143703,0). Using Maple, the eigenvalues for the hr-vaccine-type HPV equilibrium *E* ^1^ described by Jacobian matrix *J*(*E* ^1^) were calculated to be

(A.2) τ11=−1.31748003920808<0,τ21=−0.0634672621141527<0,τ31=−0.237110360597771<0,τ41=0.169597275800000>0.

The presence of a positive eigenvalue then the hr-vaccine-type HPV equilibrium *E* ^1^ = (4168,26387,143703,0) is unstable.

Finally we examine the local stability of hr-nonvaccine-type HPV equilibrium *E* ^2^ = (2253,5165,0, 156043). Using Maple, the eigenvalues for *E* ^2^ described by Jacobian matrix *J*(*E* ^2^) were calculated to be

(A.3) τ12=−0.0625668812935598<0,τ22=−1.55889062829091<0,τ32=−1.29450852209553<0,τ42=−0.03539012562000<0.

The eigenvalues are negative; therefore *E* ^2^ = (2253,5165,0, 156043) is locally asymptotically stable.
